# An Unusual Case of Phagocytic Histiocytes on Peripheral Blood Smear of a Patient With Methicillin-Resistant Staphylococcus aureus (MRSA) Endocarditis

**DOI:** 10.7759/cureus.49042

**Published:** 2023-11-19

**Authors:** Maria Ali, Maliha Sumbul, Muhammad Nadeem

**Affiliations:** 1 Hematology, National Institute of Cardiovascular Diseases, Karachi, PAK; 2 Hematology, National Institute of Blood Diseases and Bone Marrow Transplantation, Karachi, PAK

**Keywords:** hemophagocytes, ventricular septal defect, infective endocarditis, mrsa, hemophagocytic lymphohistiocytosis (hlh)

## Abstract

Hemophagocytic lymphohistiocytosis (HLH) is a rare disorder of the immune system that may rapidly progress into life-threatening complications. There is uncontrolled activation of histiocytes and phagocytic macrophages, resulting in excess secretions of cytokines that manifest into systemic hyperinflammation affecting several organs. HLH may present as primary due to underlying familial or genetic causes or as secondary due to underlying conditions such as malignancies, infections, or autoimmune disorders. This case is of a seven-year-old girl who developed culture-proven MRSA infective endocarditis. She received sensitivity-tailored antimicrobial treatment. Initially unremarkable, but later her peripheral blood smear showed the presence of large histiocyte-like cells showing hemophagocytic activity of moderate grade (two to five histiocytes with hemophagocytosis/slide) on the feathered edge of the slide. Peripheral film findings triggered further workup for HLH. Her condition later deteriorated, requiring high-dependency unit care. This case highlights the significance of maintaining a high index of suspicion with subsequent laboratory work in cases that develop cardinal diagnostic criteria for clinical manifestations of HLH. It also emphasizes reviewing the feathered edges of blood smears to prevent overlooking important morphological findings.

## Introduction

Hemophagocytic lymphohistiocytosis (HLH) is a rare ailment of the immune system that is provoked by various stimuli, such as cancer or infection. It causes excessive secretion of cytokines owing to unchecked activation and proliferation of macrophages and lymphocytes, subsequently leading to multiorgan failure with endothelial damage [1-2]. It occurs when the activity of histiocytes and lymphocytes becomes uncontrolled, which starts phagocyting the body’s own cells along with pathogens. Correspondingly, several body tissues can be invaded and attacked by these overactive cells.

HLH has the potential to precipitate into severe complications or even progress to a fatal outcome. It can be categorized into two forms: a primary genetic form and a secondary form that develops in conjunction with various conditions such as infections, autoimmune disorders, and malignancies. At least five out of the following eight conditions must be present to conform with current criteria: fever, cytopenia affecting two blood cell lines, hypertriglyceridemia and/or hypofibrinogenemia, hyperferritinemia (levels greater than 500 µ/L), hemophagocytosis, elevated soluble interleukin-2 receptor (CD25), decreased natural killer-cell activity, and splenomegaly [3].

Considering the possibility that all diagnostic criteria may emerge over time, it may be prudent to monitor patients for the development of new symptoms. Occasionally, it may appear with symptoms other than the typical ones described earlier, including liver failure, lung problems, skin manifestations, neurological abnormalities, and visual symptoms [4].

Morphological findings of hemophagocytic histiocytes are predominantly evident in bone marrow aspirate films or tissue biopsies, and they are seldom observed in peripheral blood films. However, when it does appear in peripheral blood smears, it is typically associated with a high risk of fatality [5]. We present a case involving a seven-year-old girl who had methicillin-resistant Staphylococcus aureus (MRSA) infective endocarditis and subsequently displayed characteristic hemophagocytic activity on her peripheral blood smear.

## Case presentation

The present case is of a seven-year-old girl who had a history of unrepaired ventricular septal defect, weighed 15 kg, and was admitted to the hospital through the emergency department. She reported experiencing intermittent fever up to 38.8°C, vomiting, and headaches for the last week, with subsequent improvement in symptoms after taking antipyretics. There was no history of sore throat, flu, abdominal pain, loose stools, joint pain, or skin rashes. The patient's medical and surgical history did not reveal any significant events, and her birth and developmental history were unremarkable. Additionally, her vaccinations were up-to-date.

Her vitals charting showed a fever of 38.5°C, a heart rate of 100 beats per minute, and a respiratory rate of 35 breaths per minute. Abdominal examination exhibited a soft, non-tender abdomen with no palpable organomegaly. The central nervous system examination of the patient appeared normal, and on auscultation of the chest, an additional sound of grade 3/6 murmur was audible in the left parasternal space.

Blood cultures, ultrasound abdomen, and echocardiography, along with routine investigations, were ordered by the primary care team. The ultrasound abdomen showed mild splenomegaly of 9.5 cm and mild pleural effusion with an underlying collapsed lung. A CT scan of the chest revealed cardiomegaly, ventricular septal defect, pulmonary hypertension, findings of right lower lobe infarcts, bronchiectasis changes, pulmonary edema, and pleural effusion. Blood cultures repeatedly grew MRSA on two separate occasions, and she was started on vancomycin and meropenem. Transthoracic echocardiography showed vegetations of the septal leaflet of the tricuspid valve (15x9 mm), which was subsequently reviewed after two weeks, showing enlargement in vegetations (20x10 mm). The results of blood cultures and findings of the transthoracic echocardiogram conformed to Duke's criteria for the diagnosis of infective endocarditis.

**Figure 1:**
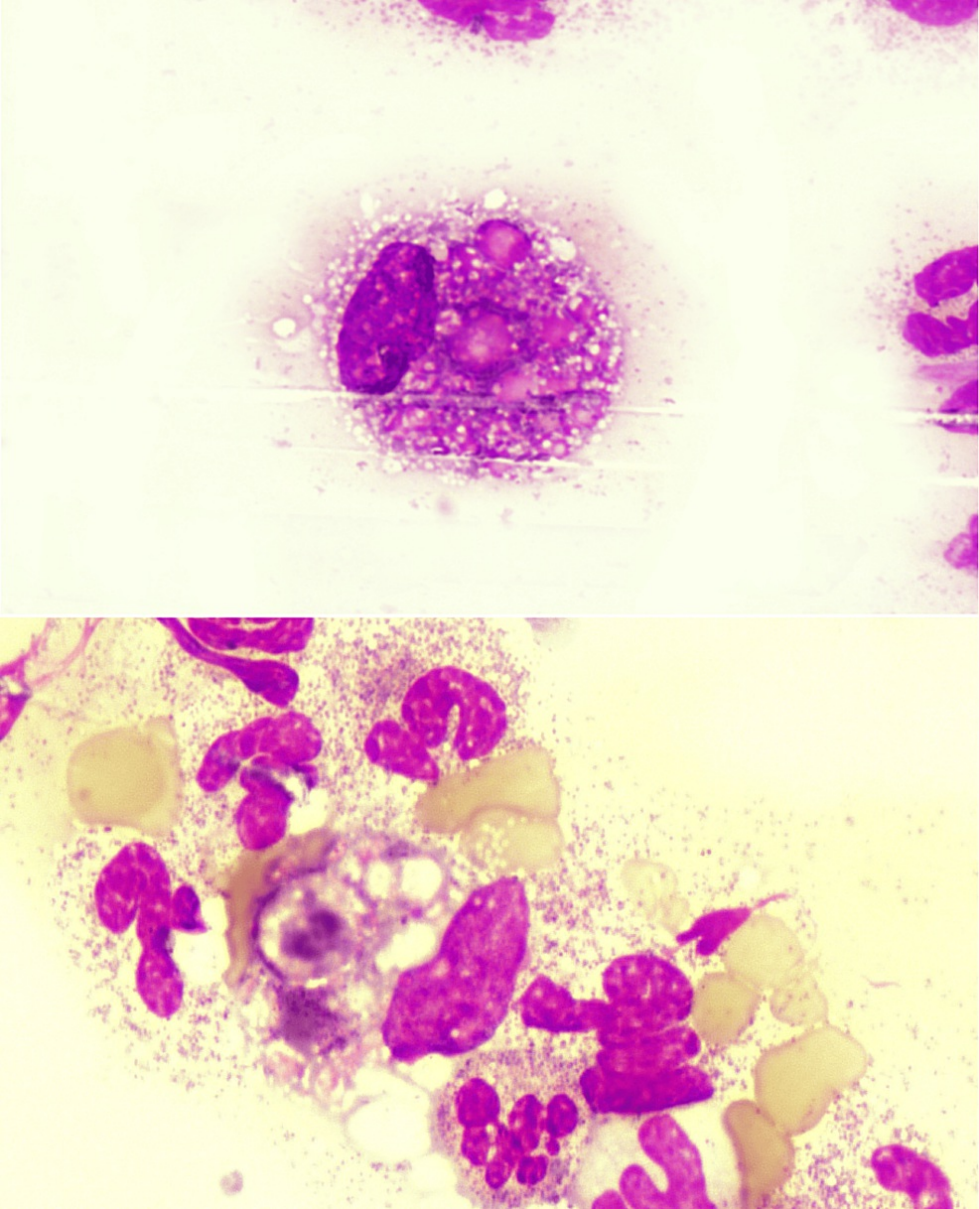
Feathered edges of the peripheral blood smear on examination at high magnification (100x) reveal histiocytes exhibiting characteristics of hemophagocytosis, engulfing leukocytes, and mature erythrocytes

Her condition deteriorated during the hospital stay, despite the treatment. She received multiple transfusions during her stay. She gradually developed a continuous fever with oral thrush, for which she was given fluconazole. Peripheral blood did not show any significant abnormalities in the beginning. However, during the last day of her 20-day stay in the hospital, her blood smear showed the presence of histiocytes showing hemophagocytic activity of moderate grade (two to five histiocytes with hemophagocytosis/slide) on the feathered edge of the smear (Figure 1). She was found to have a hemoglobin of 89 g/L, a hematocrit of 25%, a mean corpuscular volume of 78 fL, a mean corpuscular hemoglobin of 27.8 pg, a WBC of 19 x109/L, and a platelet count of 25 x109/L. Further workup revealed serum ferritin of 1470 ng/mL and triglycerides of 215 mg/dL (Table 1). Despite the importance of fibrinogen as a crucial diagnostic marker, it was not feasible to perform the test. The collected sample deteriorated, rendering it unsuitable for testing, thereby preventing the assessment of fibrinogen levels.

**Table 1:** The workup of laboratory investigations during the course of hospitalization SGPT, glutamic-pyruvic transaminase; SGOT, glutamic-oxaloacetic transaminase; GGT, gamma-glutamyl transferase; ALP, alkaline phosphatase; PT, partial thromboplastin time; aPTT, activated partial thromboplastin time; CRP, C-reactive protein; ESR, erythrocyte sedimentation rate; RA factor, rheumatoid factor

Investigations	Units	Initial values	Values at the end of the hospitalization period
Hemoglobin	g/L	63	85
Leukocytes	x10^9^/L	7.1	19
Platelets	x10^9^/L	72	25
Reticulocyte count	%	0.5	0.7
Creatinine	mg/dl	0.4	0.7
CRP	mg/dl	11.3	8.8
Sodium	mEq/L	131	125
Potassium	mEq/L	4.0	3.5
Chloride	mEq/L	102	92
PT	Sec	12.8	14.2
aPTT	Sec	33.7	27.5
Total bilirubin	mg/dl	0.7	0.9
GGT	U/L	15	22
SGPT	U/L	15	22
SGOT	U/L	51	35
ALP	U/L	83	113
ESR		65	
RA factor		<8	
Serum ferritin	ng/ml		1470
Triglycerides	mg/dl		215
Serum fibrinogen	mg/dl	NA	NA

Her HScore was calculated without fibrinogen levels and was found to be 178 points (optimal cutoff of 169) with a 54-70% probability of hemophagocytic syndrome, with little modification that hemophagocytosis was evident on a blood smear rather than a bone marrow aspirate. Her condition deteriorated, and she developed respiratory distress requiring ventilator support. Her condition did not show any improvement despite all the critical care support, and she expired in the next 24 hours.

## Discussion

MRSA is one of the rarest causes of secondary HLH. The finding of the hemophagocytic picture of the peripheral blood smear itself is one of the rarest observations that triggered further HLH-related investigations in our case. Secondary HLH is commonly related to infections or malignancy; however, it is also found to be associated with autoimmune disorders and occasionally with metabolic disorders [6]. The overactivity of histiocytes results in cytokine storms, which leads to an intensified inflammatory state within the body. Hemophagocytosis is an explicit attribute of HLH, yet it may not be present in some cases, or it may not be present in the earlier course of the disease but later becomes evident [7], as we observed in our case.

Soluble IL2 (sIL2) receptor, though a crucial diagnostic test for HLH, was originally reported to have a very high sensitivity of 93% and specificity of 100% (IL2 >2400) and is seldom used in clinical diagnosis [8]. Likewise, even though the presence of the sIL2 receptor and decreased NK activity are given particular importance in the HLH-2004 review as diagnostic criteria [9], due to a lack of availability, these tests were not performed in our patient.

HLH could be very challenging to diagnose; however, it should be considered and investigated in antimicrobial-resistant infections [9]. Cytological evidence of hemophagocytosis, although considered highly reliable, is subjective and can vary depending on the observer, making it susceptible to being overlooked by inexperienced clinicians [10]. In addition to conventional HLH 2004 and modified 2009 criteria, the HScore calculator is a great tool for faster diagnosis. HScore has an edge over traditional criteria in that it is less confining, and the probability of a positive diagnosis goes higher with an increased score [11]. Fardet et al. developed the "HScore," a diagnostic system tailored for identifying reactive hemophagocytic lymphohistiocytosis (HLH) [12]. This approach involves assigning specific values to clinical and laboratory criteria. By incorporating both clinical and laboratory factors, this scoring system offers a more thorough and meticulous perspective on the probability of reactive HLH in patients. Correspondingly, in our situation, despite the unavailability of serum fibrinogen results, the H score calculator provided a probability score higher than the optimal cut-off value. This finding enhanced our clinical suspicion and provided additional support for the diagnosis.

Using a standardized treatment modality may not be appropriate due to variations in the underlying causes, which is particularly important in cases of secondary HLH where a treatment strategy that targets the underlying trigger is necessary [4]. In infection-related HLH, the main focus of treatment is on controlling the cytokine storm leading to hyperinflammation and treating the causative pathogen. Antimicrobial therapy alone may not be adequate, requiring steroids to control hyperinflammation; however, immunosuppression itself may deteriorate the patient’s condition.

## Conclusions

A meticulous review of the feather edge of the blood smear is of paramount significance due to the displacement of histiocytes and other larger cells toward this region during the preparation of the blood smear. Consequently, it is important to thoroughly examine this area for accurate analysis so that such findings are not left unnoticed. The presence of HLH in conjunction with MRSA endocarditis serves as an indicator of the disease's severity. It is crucial to maintain a high level of suspicion and utilize valuable laboratory investigations along with other useful tools such as the H score, HLH 2004, and modified HLH 2009 criteria for diagnosis. The therapeutic modality must focus on specifically addressing the underlying primary pathology.
